# Quorum Quenching Approaches against Bacterial-Biofilm-Induced Antibiotic Resistance

**DOI:** 10.3390/antibiotics13070619

**Published:** 2024-07-03

**Authors:** Patrizia D’Aquila, Elisabetta De Rose, Giada Sena, Angelo Scorza, Bonaventura Cretella, Giuseppe Passarino, Dina Bellizzi

**Affiliations:** 1 Department of Biology, Ecology and Earth Sciences, University of Calabria, 87036 Rende, Italy; patrizia.daquila@unical.it (P.D.); elisabetta.derose@unical.it (E.D.R.); giada.sena@unical.it (G.S.); giuseppe.passarino@unical.it (G.P.); 2 Villa Ermelinda, Progetto Terza Età, 88842 Cutro, Italy; angeloscorza10@gmail.com (A.S.); dottcretella@inwind.it (B.C.)

**Keywords:** antibiotic resistance, biofilm, Quorum Sensing, Quorum Quenching

## Abstract

With the widespread phenomenon of antibiotic resistance and the diffusion of multiple drug-resistant bacterial strains, enormous efforts are being conducted to identify suitable alternative agents against pathogenic microorganisms. Since an association between biofilm formation and antibiotic resistance phenotype has been observed, a promising strategy pursued in recent years focuses on controlling and preventing this formation by targeting and inhibiting the Quorum Sensing (QS) system, whose central role in biofilm has been extensively demonstrated. Therefore, the research and development of Quorum Quenching (QQ) compounds, which inhibit QS, has gradually attracted the attention of researchers and has become a new strategy for controlling harmful microorganisms. Among these, a number of both natural and synthetic compounds have been progressively identified as able to interrupt the intercellular communication within a microbial community and the adhesion to a surface, thus disintegrating mature/preformed biofilms. This review describes the role played by QS in the formation of bacterial biofilms and then focuses on the mechanisms of different natural and synthetic QS inhibitors (QSIs) exhibiting promising antibiofilm ability against Gram-positive and Gram-negative bacterial pathogens and on their applications as biocontrol strategies in various fields.

## 1. Introduction

Quorum Sensing (QS) is a global gene regulation mechanism based on bacterial cell-to-cell communication achieved through the release, detection, and response of extracellular signaling molecules called autoinducers (AIs) [1]. It can be considered an adaptive response to the high cellular density at which the cumulative production of AIs occurs, enabling detection and response through a specific transcriptional regulator. In Gram-positive and Gram-negative bacteria, different types of QS systems have been widely described, and different classes of AIs have been identified [2]. QS allows bacteria to perceive their surroundings, regulate their density and behavior, and optimize the use of available nutrients, giving them the ability to live as multicellular organisms. What is more, it makes the cell community manage self-competition as well as collectively interact with their host. QS regulates several biological processes including bioluminescence, sporulation, competence, antibiotic production, and virulence factor production by pathogenic bacteria, as well as the formation and maintenance of biofilm, the complex, three-dimensional structures representing a long-established survival mechanism for bacteria [3]. The high cell density that is created in the biofilm makes it the ideal condition for QS regulation. In fact, numerous QS-dependent genes code for the biosynthesis of exopolysaccharides that directly participate in the architecture of mature biofilms. Biofilms play a fundamental role in pathogenesis as they can evade the human immune response, and bacteria forming them exhibit resistance to treatment with various antibiotics, making fighting these infections even more challenging, leading to prolonged illness and contributing significantly to high morbidity and mortality rates [4]. The important role played by biofilms in microbial infections is more than relevant if we consider that the National Institutes of Health (NIH) revealed that among all microbial and chronic infections, 65% and 80%, respectively, are associated with biofilm formation. These infections are contracted in hospital settings by patients undergoing long-term hospitalization or in close contact with medical devices and implants on which biofilms form, increasing the risk of infection during surgeries or other medical procedures. They are colonized with extreme efficiency by microorganisms of the skin flora, including Gram-positive bacteria such as *Staphylococcus aureus*, which use them as “entry routes” to the patient’s internal organs, causing infections with often very serious outcomes [5].

Starting from the above consideration, recently, control strategies have been developed to activate so-called Quorum Quenching (QQ), thus providing new possibilities for dealing with infectious diseases and overcoming and solving the problem of microbial resistance. QQ interferes specifically with the QS system of bacteria, hindering the exchange of information between them and reducing the expression level of hazard factors. It may be mainly achieved by inhibiting the production of AI molecules, by inhibiting AI detection through the inactivation of the receptors, or by enzyme-catalyzed degradation or modification of the AI molecules. Different classes of QQ enzymes as well as QQ inhibitors have been identified so far, thus highlighting how they exhibit substrate specificities [6].

The present paper aims to review the current knowledge regarding the implication of the biofilm process in the phenomenon of antibiotic resistance and the QQ strategies. The first section describes the stages of biofilm formation and its structural elements as well as its intrinsic characteristics that are the basis of biofilm-induced antibiotic resistance. The next section reviews the mechanisms of inhibition of the QS signaling pathway for controlling biofilm formation and then focuses on the antibiofilm mechanisms of different natural and synthetic QS inhibitors. The final section reports the strategies which are currently being adopted as biocontrol solutions in various fields.

## 2. Biofilm and Antibiotic Resistance

Biofilm is an aggregation of microbial cells attached to biotic or abiotic surfaces embedded in a self-produced extracellular polymeric substance (EPS). The shift in bacteria from a free-swimming planktonic state to a biofilm-making sessile encompasses multiple stages: attachment to a surface, microcolony formation, biofilm maturation, and bacterial dispersion [7]. During the initial stage of biofilm formation, planktonic cells interact very briefly and transiently with a surface through Van der Waals forces by using flagella, pili, and fimbriae, depending on favorable environmental conditions. The gradual buildup in cellular cAMP levels and a gradual corresponding increase in type IV pili in the surface-associated cells make the attachment irreversible, and the cells start the production of an extracellular polymeric matrix composed of polysaccharides, proteins, and extracellular DNA (eDNA) that clump together, forming microcolonies [8].

EPS-embedded growing cells organize themselves into a three-dimensional structure, in which they interact, communicate, and cooperate through the complex communication system of QS. Lastly, intrinsic factors of the bacterial community, namely genetic changes, overpopulation, and intense competition for nutrients, as well as environmental perturbations like changes in nutrient concentrations and temperature, oxygen deficiency, and metabolite accumulation, lead to the dispersion of bacterial cells that return to a planktonic state [9].

The intrinsic properties of biofilms provide the microbial community with characteristics towards antibiotic resistance. It is interesting to consider Chen and Wen’s perspective, according to which bacterial biofilm is a particular kind of persistent bacterial infection [10]. Besides its structural role in biofilm, EPS represents a physical barrier for a biofilm since it hinders or reduces the penetration of antimicrobial compounds in the innermost layers. An example, ionic interactions between antimicrobial drugs and the EPS matrix may limit their penetration. The negative charge of polysaccharides effectively blocks the penetration of positively charged antibiotics such as aminoglycosides [11]. In addition, the EPS barrier leads to an incomplete penetration of antibiotics, and therefore, the cells in the biofilm layer encounter sub-inhibitory concentrations of the drug, becoming tolerant [12,13]. It has also been reported that extracellular DNA (eDNA), a key constituent of the EPS matrix, by acting as a metal cation chelator, activates the PhoPQ/PmrAB systems that control various genes involved in nutrient utilization, metal homeostasis, acid pH tolerance, and virulence in *Pseudomonas aeruginosa* [14]. The heterogeneity of the microenvironment within biofilm, characterized by a progressive gradient of pH, oxygen, and nutrients from the top to the bottom of the biofilm, also contributes to the acquisition of drug-resistant phenotypes. Andrè and coll. reported that pathogens benefit from hypoxia, which has been proven to activate various mechanisms of pathogen virulence [15]. An acidic microenvironment promotes faster bacterial evolution toward elevated antibiotic resistance, as evidenced by promoting the selection of stable genetic mutations and increasing the expressions of multiple biofilm- and virulence-related genes [16]. Further, in reduced levels of nutrients and oxygen, cells globally decrease their bacterial metabolic and growth rates, thus promoting the emergence of persister cells, a subpopulation of transiently slow-growing or growth-arrested cells that are tolerant to antibiotics and able to survive for extended periods in the absence of nutrients and lethal stresses [17,18]. The presence of persister cells can result in the recalcitrance and relapse of persistent bacterial infections, and it has been linked to an increase in the risk of the emergence of antibiotic resistance [19]. The increased resistance capacity of biofilms is also attributable to the upregulation of efflux pumps in response to antimicrobial exposure, thereby extruding multiple classes of antimicrobial compounds into the extracellular environment. A few efflux pump genes were found upregulated, mainly in the upper layer of biofilms. These include *AcrAB-TolC* in *Escherichia coli*, *AdeFGH* in *Acinetobacter baumannii*, PA1874-1877 and *MexAB-OprM* in *P. aeruginosa*, Resistance–Nodulation–cell Division (RND) in *Burkholderia cenocepacia*, and *AcrD* in *Salmonella enterica* [20,21,22,23]. Efflux pumps are also implicated in the secretion of QS molecules and thereby indirectly modulate gene expression remodeling underlying cell-to-cell adhesion, EPS and virulence factor production, and biofilm formation [24]. Lastly, within a biofilm, mobile genetic elements, including plasmids, transposons, and integrons, may facilitate the spread of resistance genes among the microbial community through horizontal gene transfer [25].

## 3. Anti-QS Approaches to Overcome Biofilm Resistance

Over the past two decades, several agents of diverse origins have been identified to suppress biofilm formation through the inhibition of the QS signaling pathway by exerting inhibitory effects at multiple levels. According to their target, these agents are generally grouped into three categories: inactivators of signaling molecules, inactivators of signaling receptors, and inhibitors of signaling cascade, through the mechanisms described below.

### 3.1. Inactivation of Signaling Molecules

Since inter- and intra-species bacterial communication is sensed, maintained, and powered by QS mechanisms and represents a crucial step for biofilm formation, an effective strategy to interrupt this communication is represented by the use of inhibitors capable of suppressing the synthesis of, inactivating, or degrading the AI signaling molecules. Studies on *P. aeruginosa* revealed that (z)-5-octylidenethiazolidine-2,4-dione (TZD-C8) significantly downregulates the expression of LuxI-type acyl-homoserine lactone synthases by interfering with both the Pseudomonas Quinolone Signal (PQS) and 3-oxo-C12-HSL signaling pathways [26]. In vitro studies also reported that subminimal growth-inhibitory concentrations of some macrolide antibiotics are able to reduce N-acyl homoserine lactone (AHL) synthesis in *P. aeruginosa*. Tateda and coll. revealed that azithromycin interferes with the synthesis of autoinducers by reducing the concentration of 3-oxo-C12-HSL and C4-HSL by 94 and 72%, respectively, and the expression of the transcriptional activator genes *lasR* and *rhlR* and of the two autoinducer synthase genes *lasI* and *rhlI* [27]. Subminimal growth-inhibitory concentrations of erythromycin were found to suppress the synthesis of homoserine lactone autoinducers as well as the production of hemagglutinins, proteases, and hemolysins [28].

Alternatively, AHL production could be blocked by targeting its two precursors, which are S-adenosylmethionine (SAM), an amino donor for the generation of the homoserine lactone ring moiety, and acyl carrier protein (ACP) for the acyl side chain of the AHL signal. Various analogues of SAM, such as S-adenosylhomocysteine, S-adenosylcysteine, and sinefungin, were demonstrated to be potent inhibitors of AHL synthesis [29]. A low concentration of azithromycin inhibits the expression of numerous genes belonging to the SAM synthesis pathway which, in turn, leads to a decrease in AHL production by LasI and RhlI [30]. What is more, triclosan reduces AHL synthesis by inhibiting the precursor of enoyl-ACP reductase [31].

A wide range of molecules have been progressively identified to inhibit AI-2 synthesis via the downregulation of the LuxS enzyme across a wide spectrum of bacteria. Next to S-homoribosyl-L-cysteine and L-homocysteine, sinefungin, a variety of halogenated furanones, and natural compounds like surfactin were recognized to downregulate the *luxS*, *pfs*, and *speE* genes, with a significant reduction in AI-2 production in several bacterial species, such as *Streptococcus pneumoniae*, *Streptococcus suis*, and *Staphylococcus epidermidis* [32,33,34,35].

Ultimately, the inactivation or degradation of signal molecules by AHL quenching represents another effective QQ strategy interfering with bacterial communication. In this context, three different classes of enzymes have been progressively discovered and classified according to their mechanism of action. These enzymes, their substrates, and relative effects on biofilm inhibition are reported in Table 1.

AHL lactonase hydrolyzes the ester bond of the homoserine lactone ring [71]. Most of the AHL-degrading enzymes are lactonases belonging to the metallo-lactamase superfamily [72]. Identified for the first time by Dong and coll. in *Bacillus* species isolated from soil samples, these enzymes have been progressively discovered in several other bacterial species, including *A. tumefacens*, *Bacillus stearothermophilus*, *K. pneumoniae*, *Rhizobium* sp., *Mycobacterium avium*, *Muricauda olearia*, and *V. cholerae* [6,36,38,43,45,46,51]. Most of the known AHL-lactonases have high affinity and stability at high temperatures and do not display any preference for the length of the carbon acyl side chain attached to the lactone ring [73].

Still identified in only a limited number of bacterial species, AHL acylases catalyze the degradation of the AHL amide bond, generating free fatty acids and a lattone ring [52,54,60,74,75]. It has been suggested that acylases degrade AHL with a long side chain than with a short side chain [76]. Lastly, several AHL oxidoreductases are known from various bacteria, reducing or oxidizing the acyl chain of the AHL, thus inhibiting the specific binding of the autoinducer to its receptor [65,66,69]. Only a few enzymes degrading or modifying AI-2 have been currently identified. Xavier and coll. identified in *E. coli* the kinase LsrK responsible for the phosphorylation of AI-2 as well as LsrG, potentially involved in the degradation of phospho-AI-2 [77,78]. In addition, since AI-2 in its dephosphorylated form is unable to bind to its receptor, and therefore the signaling cascade does not begin, it follows that many compounds exerting inhibition activity against LsrK have been progressively identified [79,80,81]. Furthermore, high concentrations of imidazole, a furan carbocyclic analogue of AI-2, decrease the antibiotic resistance of a clinical ampicillin-resistant *E. coli* strain by significantly downregulating the transcription of the *lsrR* gene that, in turn, reduces the function of AI-2 [82]. More recently, by using a metagenomic approach, several non-toxic biomolecules were identified. Particularly, it was demonstrated that the oxidoreductase QQ-2, simultaneously interfering with AI-2 and AHL, reduces 4-hydroxy-2,3-pentanedione-5-phosphate (P-DPD, a C5-phosphorylated derivative of the open AI-2 form) to 3,4,4-trihydroxy-2-pentanone-5-phosphate, namely, an inactive AI-2 derivative [70].

### 3.2. Inactivation of Signaling Receptors

In the last ten years, a few QS inhibitors competing with autoinducers for binding to their receptors have been discovered by computational docking studies as well as high-throughput screening (HTS) of small-molecule libraries, mainly in *P. aeruginosa*.

Flavonoids such as naringenin were demonstrated to directly bind the receptor LasR, thus preventing LasR/RhlR DNA binding, reducing biofilm formation, and repressing many other QS-related effects [83,84]. Similar QQ mechanisms were identified for many other natural compounds, including ortho-vanillin, embelin, and piperine, as well as for the antidiabetic drugs dipeptidase inhibitor-4 (DPI-4) sitagliptin and omarigliptin [85,86,87,88]. In addition, in vitro and molecular docking assays demonstrated that subinhibitory concentrations of several aminoglycoside drugs, including amikacin, gentamicin, kanamycin, neomycin B, paromomycin, and netilmicin, display strong binding properties with the LasR protein and, thus, exhibit antibiofilm action against *P. aeruginosa* [89]. More recently, Manson and coll., by screening a 25,000-compound library, discovered eight new robust and effective antagonists of LasR [90].

Numerous structural analogues of AHL were identified as antagonists of QS receptors to compete or interfere with the signal of autoinducers. In this context, several data demonstrated that the length of the acyl chain, as well as the introduction of an unsaturated bond close to the amide linkage of AHL, has a significant impact on its binding activity, so that AHL analogues with a long acyl side chain represent efficient inhibitors [91,92]. In addition, many natural compounds, including catechin, nakinadine B, flavonoids, and furanones, as well as many synthetic AHL analogues, were identified to exert a competitive inhibition on autoinducer receptors in several bacteria [84,93,94,95,96,97]. What is more, by using a multivalent scaffold approach, a chemical antagonist probe for Lsr-type AI-2 receptors in *Salmonella typhimurium* and an imaging agent for bacterial species utilizing Lsr-type AI-2 receptors were identified [98]. The antagonistic effect of several AI-2 synthetic analogues was recognized in *Vibrio harveyi* [99].

### 3.3. Inhibition of Signaling Cascade

The best-characterized mechanism of inhibition of the QS signaling cascades involves the use of targets blocking the downstream response regulator AgrA in *S. aureus*. Many compounds, such as Azan-7, bumetanide, savrin, and staquorsin, have been discovered to strongly reduce the AgrA-DNA complex formation at the P3 promoter region involved in the regulation of RNAIII transcription, thus preventing virulence gene upregulation and biofilm formation [100,101,102]. A few synthetic molecules have been discovered to act as antagonists of LuxR-type proteins, such as many autoinducer analogs. The TraR regulator response of *Agrobacterium tumefaciens* was demonstrated to be antagonized by analogs of the autoinducer 3-oxo-octanoyl-homoserine lactone [103]. Virstatin, a small molecule that prevents the expression of cholerae virulence factors, is able to repress the expression of AnoR that is a positive regulator of the LuxI-like synthase AnoI in *Acinetobacter nosocomialis*, leading to decreased synthesis of N-(3-hydroxy-dodecanoyl)-L-homoserine lactone (OH-dDHL). Low levels of this compound affect the signaling cascade and reduce biofilm formation and motility [104]. What is more, PAβN, an efflux pump inhibitor, reduces the extracellular accumulation of QS signaling molecules such as N-3-oxo-dodecanoyl-l-homoserine lactone and N-butyryl-l-homoserine lactone and significantly decreases the relative expression of the QS cascade (*pqsA*, *pqsR*, *lasI*, *lasR*, *rhlI*, and *rhlR*) and QS-regulated type II secretory genes *lasB* (elastase) and *toxA* (exotoxin A) in *P. aeruginosa* clinical isolates, thus inducing a reduction in bacterial virulence [105]. In *E. coli* and *Salmonella*, inhibitors of the transcription of the SdiA regulator protein, such as extracts of *Melia dubia* seeds and fructose-furoic acid, attenuate the expression of virulence factors by blocking the binding of AHLs to SdiA [106]. A phosphate ester derivative of chrysin exhibits higher anti-virulence activity by acting as a potent QS inhibitor of *P. aeruginosa*. It binds to the QS regulator LasR, thus abrogating its DNA-binding capability [107].

**Figure 1:**
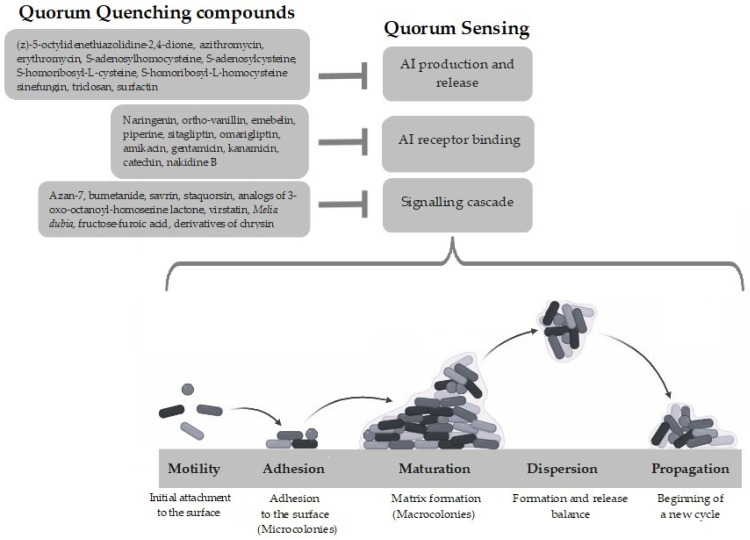
Schematic diagram of the different levels at which Quorum Sensing acts in regulating biofilm formation and compounds effective in its inhibition. AI: autoinducer. Figure made using Biorender.com.

In Figure 1, the three different anti-QS approaches described above are schematized.

## 4. Quorum Quenching Compounds

Regarding their origin, QSIs can be distinguished into compounds of natural or synthetic provenance. Many QQ agents identified to date were isolated from various natural sources, such as plants, marine organisms, and living organisms.

### 4.1. Plant-Derived Anti-Biofilm Compounds

Plants have been known for centuries to be sources of a plethora of bioactive compounds with antioxidant, anticarcinogenic, antiallergenic, anti-inflammatory, antimutagenic, and antimicrobial activities, and it is no coincidence that phytochemicals represent the basis of traditional medicinal practices [108,109,110]. As part of the characterization of the antimicrobial properties of these compounds, referred to as phytochemicals, numerous studies have highlighted how the majority exert their action by significantly inhibiting the process of bacterial biofilm formation and the QS signaling system. Among these, we mention alkaloids, lectins, polyacetylenes, polyphenols, and terpenoids, of which a summary of the main compounds is shown in Table 2.

In recent years, special attention has been given to Essential Oils (EOs), volatile secondary metabolites of aromatic plants and spices that give them their characteristic and distinctive smell or taste, that exhibit recognized antimicrobial properties. In this frame, several studies have revealed how EOs can inhibit biofilm formation through different mechanisms. *Calamintha nepeta* (L.) *Savi* ssp. *nepeta*, *Clinopodium nepeta* L., *Cinnamomum verum*, *Cinnamomum zeylanicum*, *Citrus bergamia*, *Citrus limon*, *Citrus reticulata*, *Eugenia caryophyllata*, *Foeniculum vulgare* subsp. *piperitum*, *Laurus nobilis* L., *Lippia alba*, *Myrtus communis* L., *Origanum vulgare* spp., *viridulum Ocimum basilicum*, *Prangos trifida*, *Petroselinum crispum*, *Salvia officinalis* L., *Salvia Rosmarinus*, *Satureja hortensis*, *Thymus daenensis*, and *Thymus vulgaris* are just a few oils whose anti-biofilm properties have been demonstrated in both Gram-negative and Gram-positive bacteria as well as in multidrug-resistant clinical strains [137,138,139,140,141,142,143].

The biological activity of each EO has been mostly attributed to the hydrophobic nature of oils as well as to the action of their principal components, which are represented mainly by terpenes and terpenoids as primary components, and polyphenols [144]. It has been demonstrated that most of these compounds interact with the lipid bilayer and accumulate in the cell membrane, leading to a progressive loss of intracellular molecules such as nucleic acids and proteins [145,146]. Recent reports have also highlighted that EOs induce a profound altering of the global methylation levels of adenine and cytosine residues located in the genomes of both pathogenic and non-pathogenic bacterial strains, thus hypothesizing that they induce a massive epigenetic remodeling that, in turn, profoundly changes the expression of genes involved in cooperative behaviors, cell-to-cell adhesion and communication, and virulence and hinders the strategies adopted for the survival of the bacterial community [147].

### 4.2. Marine-Derived Anti-Biofilm Compounds

Also, the marine environment, with algae, invertebrates, sponges, and corals, represents a rich source of natural bioactive molecules; thus, in the last few years, their antibiofilm activity has been progressively identified. The inhibitory effects on biofilm formation exerted by extracts or derivatives from seaweeds and microalgae were recently reviewed by Behzadnia [148]. As an example, extracts from *Westiellopsis prolifica* inhibit biofilm formation and prevent cell adhesion against Gram-positive (*S. aureus, Bacillus subtilis*, and *Streptococcus* spp.) and Gram-negative bacteria (*Shigella* sp., *Proteus* sp., and *P. aeruginosa*), as well as fungi (*Aspergills niger* and *C. albicans*) [149]. Crude extracts from *Arthrospira platensis* (cyanobacteria) and *Polysiphonia scopulorum* (Rhodophyta) show strong antimicrobial and antibiofilm activity and are responsible for a significant downregulation of genes that play pivotal roles in surface attachment, biofilm formation, and the overall stability of the biofilm architecture in *P. aeruginosa* [150]. Similarly, *Chlamydomonas* extract inhibits the QS pathway, thus affecting *P. aeruginosa* pathogenicity and biofilm morphology and thickness [146]. Extracts from *Cladostephus spongiosus* (Phaeophyta) inhibit hyphal growth and biofilm formation via the downregulation of the expression of hyphal-specific genes and virulence factors in *Candida* sp. [151]. In addition, algal bioactive compounds have been progressively identified as anti-biofilm agents by mainly interfering with the expression of genes involved in QS signaling. Among these, we may mention algal polysaccharides, such as alginate, laminaran, and fucoidan, algal carotenoids, including zeaxanthin and lutein, algal lipids, of which α-linolenic acid, monogalactosylmonoacylglycerol, palmitoleic acid, spirulina, sulfoquinovosyldiacylglycerol, and sulfoquinovosylmonoacylglycerol are just some examples, as well as algal phlorotannins, that were found to reduce cell proliferation, motility, and adhesion and interfere with the synthesis of exopolysaccarides and virulence factors [152,153,154,155,156,157,158,159,160].

Furthermore, a few compounds from marine sponges were proven to have antibiofilm activity. These include the bromoageliferin from *Agelas* spp., the darwinolide from *Dendrilla membranosa*, the diterpene alkaloid(-)-ageloxime D from *Agelas nakamurai*, the tryptamine derivative bufotenine from *Paramuricea clavate*, and Synoxazolidinonones from *Synoicum pulonaria* [161]. QS inhibition associated with biofilm disruption was observed in *Vibrio* spp. treated with a marine actinomyces extract [162].

### 4.3. Microbial-Derived Anti-Biofilm Compounds

Next to the previously described enzymes involved in the inactivation or degradation of QS autoinducers, namely lactonases, acylases, and oxidoreductases, and to the well-characterized antibiotics obtained from microorganisms, such as benzylpenicillin, cephalosporins, and gentamicin, many secondary microbial metabolites have been progressively identified to exert QQ activity [163]. Walkmycin C, a histidine kinase (HK) inhibitor produced by *Streptomyces* sp., displays anti-biofilm activity against *Streptococcus mutans* [164]. In addition, alnumycin D, granaticin B, kalafungin, medermycin, collismycin C, napyradiomycin, hygrocin C, 8-O-metyltetrangomycin, panglimycin D, and streptorubin, isolated from *Streptomyces* sp. exhibit promising antibiofilm activities against *S. aureus* and methicillin-resistant *S. aureus* (MRSA) [165,166,167,168,169].

Several microbial-produced diffusible-signal factors (DSF), which are cis-2-unsaturated fatty acids identified in a range of bacterial pathogens, including *Xanthomonas*, *Enterobacter*, *Thiobacillus*, *Leptospirillum*, *Stenotrophomonas*, *Burkholderia*, *Achromobacter*, *Yersinia*, *Methylobacillus*, *Pantoea*, and *Cronobacter*, are able to disperse biofilm formation in a range of Gram-negative and Gram-positive bacteria and modulate virulence gene expression, reverse persistence, increase microbial metabolic activity, and significantly enhance the antibacterial activity of conventional antimicrobial agents [170,171,172,173]. Carolacton, a macrolide keto-carboxylic acid produced by the myxobacterium *Sorangium cellulosum*, by interfering with PknB-mediated signaling in growing cells, significantly inhibits *S. mutans* biofilm formation [174]. Similarly, other secondary metabolite compounds, including the skyllamycin family produced by *Streptomyces* sp., phenazines from both Gram-positive and Gram-negative bacteria, and promysalin isolated by *Pseudomonas putida*, were demonstrated to exert an antibiofilm effect [175].

Biosurfactants (BSs) are amphipathic bioactive compounds that are produced mostly by microorganisms on their cell surface or secreted extracellularly, and they are classified, according to the chemical structure of the hydrophilic head group, in glycolipids, fatty acids, lipopeptides, and polymers. They are powerful anti-biofilm agents that can act as biocides as well as biodispersants up to 95.9% [176,177].

Rhamnolipids, glycolipids, surfactins, and lipopepdide biosurfactants exhibit antimicrobial, anti-fungal, and anti-biofilm activity through different mechanisms of action. In vitro and ex vivo evidence reports that a biosurfactant from the entomopathogenic fungus *Beauveria bassiana* alters cell permeability and disrupts the cell membrane, effectively eradicating the biofilm of *Microsporum canis* [178]. Some BSs, including surfactin, rhamnolipid, and lipopeptide pontifactin, alter the hydrophobicity of the cell surface, interfere with cell adhesion to other microorganisms and/or surfaces, and affect the integrity of cellular membranes, thereby dispersing the biofilm and solubilizing the component of the matrix [179,180,181]. Lastly, BSs may indirectly inhibit the biofilm by interfering with QS signals. In this context, Yan and coll. demonstrated that BSs isolated from lactic acid bacteria, including *Pediococcus acidilactici* and *Lactobacillus plantarum*, affect the expression of genes involved in biofilm formation (*cidA*, *icaA*, *dltB, agrA*, *sortaseA*, and *sarA*) and interfere with the release of AI-2 signaling factor in *S. aureus* [182].

### 4.4. Antimicrobial Peptides

Antimicrobial peptides (AMPs) are a class of agents naturally occurring in animals, plants, and microbes, exhibiting antimicrobial, anti-attachment, and antibiofilm properties against both Gram-positive and Gram-negative multidrug-resistant pathogens and fungi as well [183,184]. Most AMPs are cationic, which allows their interaction with the negatively charged bacterial membranes, which permealize, thus leading to cell death [185,186].

Evidence has reported that AMPs affect biofilm formation or degradation at different stages and through the following mechanisms of action:

(i) Inhibition of bacterial attachments to surfaces: AMPs can interfere in the early stages of biofilm formation to prevent the initial adhesion of bacteria to surfaces. Arslan and coll. reported that lactoferrin suppresses the initial attachment of *Streptococcus gordonii* coaggregates [187]. By downregulating genes encoding ABC transporters involved in cell-to-surface and cell-to-cell interactions, the peptide Nal-P-113 can inhibit *Porphyromonas gingivalis* biofilm formation [188].
(ii) Disruption of the membrane potential of biofilm-embedded cells: Different kinds of bacteriocins, including nisin A and, to a lesser extent, lacticin Q, show antibiofilm activity on clinical isolates of methicillin-resistant *S. aureus* (MRSA), altering the membrane potential [189].
(iii) Permeabilization of cell membrane: Esculentins Esc (1–21) and (CSA)-13 permeabilize the cytoplasmic membrane of *P. aeruginosa* in biofilms [190,191].
(iv) Interruption of QS signaling and gene regulation: In *P. aeruginosa*, cathelicidic LL-37 and indolicidin prevent biofilm formation via the downregulation of the transcription of the Las and Rhl systems [192]. Additionally, peptide 1037 directly inhibits biofilms by reducing swimming and swarming motilities, stimulating twitching motility, and suppressing the expression of a variety of genes involved in biofilm formation [193]. The human β-defensin 3 (hBD-3) reduces the synthesis of polysaccharide intercellular adhesin (PIA) by downregulating the expression of the *icaA*, *icaD*, and *icaR* genes in *S. epidermidis* [194].
(v) Degradation of the biofilm matrix: Peptide PI decrease *S. mutans* biofilm biomass by degrading the exopolysaccharide matrix [195]. In addition, an AMP complex produced by the maggots of blowfly *Calliphora vicina* effectively counteracts the formation of *E. coli*, *S. aureus*, and *A. baumannii* biofilms by destroying the biofilm matrix [196]. Similarly, the human antimicrobial peptide hepcidin 20 reduces the mass of the extracellular matrix and alters the architecture of the biofilm in *S. epidermidis* [197].

Lastly, bacteriocins are a group of antimicrobial peptides produced by bacteria that have the potential to disrupt biofilm either by themselves or in combination with other drugs [198]. Evidence has reported that sonorensin, a bacteriocin isolated from *Bacillus sonorensis*, inhibits the biofilm growth of *S. aureus* by increasing membrane permeability [199]. The bacteriocin LFX01A from *Lactiplantibacillus plantarum* has antibiofilm activity against *Shigella flexneri* [200]. Bacteriocins produced by *Pediococcus acidilactici* exhibit antibiofilm activity through a decrease in extracellular polymeric substances (EPSs) and a reduction in cell adhesion [201]. Bacteriocin from *Lactobacillus plantarum* was effective in counteracting biofilm formation on catheters induced by bacteria such as *P. aeruginosa* and *S. aureus* [202].

### 4.5. Synthetic Compounds

A plethora of artificially designed inhibitory molecules are being synthesized to target QS and biofilm processes in bacteria. These molecules, deeply detailed by Vashistha and coll., primarily include analogues of autoinducers of QS that competitively bind the AHL receptors, thus inhibiting QS and biofilm formation [203]. Next to these analogues, over the past 15 years, several studies have highlighted the promising potential of synthetic nanostructured materials for counteracting bacterial growth. Nanoparticles (NPs) such as gold (Au), silver (Ag), copper (Cu), palladium (Pd), platinum (Pt), or zinc oxide (ZnO) have been proposed as novel potential means of fighting bacteria thanks to their ability to induce death or limit the growth of microorganisms depending on their particular physicochemical parameters, namely the shape, size, composition, and surface functionalization [204,205,206,207]. Their high surface area-to-volume ratio, high reactivity, and stability, combined with their unique physicochemical properties, allow them to interact with microbial membranes and exhibit antimicrobial effects at low concentrations by inducing oxidative stress and disrupting membrane integrity and bacterial biomolecules [206,208]. These same parameters were also found to be fundamental in conferring antibiofilm properties to NPs. More particularly, the interaction between NPs and bacteria embedded in a biofilm is modulated by the physicochemical properties of the NPs, such as their size, charge, shape, and hydrophobicity and the EPS matrix [209]. The smaller the size of the NPs, the greater their ability to penetrate the water-filled channels of the biofilm and exert antibiofilm and antibacterial effects [210,211,212]. Likewise, shapes that allow for a high surface area-to-volume ratio give NPs greater antibacterial and anti-biofilm efficacy, with rod-like NPs exhibiting more effectiveness than spherical particles in their inhibitory action [211]. In addition, the hydrophobicity of the NP surface determines its efficacy in penetrating into biofilm. Li and coll. demonstrated that NPs coated with DSPE-PEG2000 increase their hydrophilicity, improve their penetration through mucus, and effectively eradicate *Helicobacter pylori* biofilm [213]. It has also been reported that cationic particles penetrate biofilms completely, while neutral or anionic ones are unable to do so, since the EPS substances as well as the bacterial cell wall have an overall negative charge [214]. On the other hand, the viscosity and compaction of the EPS matrix, cell density, and the characteristics of the water spaces (pores) within the EPS matrix influence the speed and penetration of NPs within a biofilm [215]. A summary of the different types of nanocomposites, their characteristics, and their antibiofilm activities has been recently published [216]. NPs can exert their antibiofilm activity via a multitude of mechanisms [217,218]. The antibacterial and antibiofilm properties of silver nanoparticles (AgNPs) have been investigated in several studies. Evidence demonstrates that AgNPs fuse with the cell membrane, altering its charge and affecting cell permeability, thus ultimately leading to cell lysis [219]. These particles are also able to downregulate the production of exopolysaccharide and alginate as well as interfere with the QS system [220]. The antibiofilm effect brought out by gold nanoparticles (AuNPs), which is enhanced by the intrinsic photothermal properties of this metal, is primarily amenable to the reduction in exopolysaccharide production, the inhibition of swimming and swarming motility, and the disruption of the cell membrane followed by a metabolic deterioration [218,221,222]. Zinc nanoparticles (ZnNPs) induce cell depolarization, increase permeability, increase oxidative stress, and alter the expression of QS genes [223]. Similarly, Superparamagnetic Iron Oxide Nanoparticles (SPIONs) have been shown to reduce biofilm formation in *S. mutans* with different effectiveness depending on their surface charge [224]. Being magnetic, the presence of an external magnetic field induces these NPs to penetrate the full depth of the biofilm, thus killing bacterial cells deep inside biofilms due to vibration damage, local hyperthermia, and ROS generation [225,226].

If nanomaterials themselves act by inhibiting biofilms quite effectively through the above-mentioned mechanisms, their action is even more enhanced when they are conjugated together to form bimetallic nanoparticles (BMNPs) or coated with QS inhibitors. As an example, studies carried out on *B. subtilis*, *Proteus mirabilis*, and *S. mutans* revealed that the antibiofilm effect of mixed ZnO:MgO NPs and Zn:CuO NPs is more prominent compared to that shown by the individual NPs [227,228]. Ho and coll. modified polyacrylamide magnetic beads with the funarone-like analogue Dihydropyrrolidone (DHP), a molecule with proven QQ activity, thus revealing the effectiveness of the substrate in reducing the growth of *S. aureus* in vitro and in subcutaneous infection models [229]. Similarly, nanocarriers of many other QQ compounds, including flavonoids, AMPs, and autoinducer-degrading enzymes, gave encouraging results in promoting the dispersion of biofilms. NPCs loaded with a combination of tt-farnesol and compound 1771, with or without myricetin, fully prevent *S. mutans* and *C. albicans* dual-species biofilm formation by impeding biomass accumulation, bacterial growth, and exopolysaccharide matrix deposition [230]. AuNPs coated with the lactonase AiiA appeared to be effective in degrading K-hexanoyl-L-homoserine lactone, suppressing EPS production, and biofilm formation in multidrug-resistant *Proteus* species [231].

Lastly, lipid-coated hybrid nanoparticles (LCHNPs) were investigated for their capability of delivering antibiotics into biofilms. In vitro experiments demonstrated that LCHNPs loaded with vancomycin eradicate up to 99.99% of *S. aureus* biofilm, an effect that is not observed following treatment with vancomycin alone [232]. NPs encapsulated with ciprofloxacin and polymyxin B show complete inhibition against *E. coli* and *S. aureus* growth and *P. aeruginosa* biofilm [233,234]. Tobramycin and an alkylquinolone QS inhibitor encapsulated in squalenyl hydrogen sulfate nanoparticles (SqNPs) allow for the complete eradication of biofilm at a 16-fold lower concentration than tobramycin alone [235]. Hybrid nanospheres containing gentamicin and QQ acylase exhibit a significant inhibitory effect on the formation of *P. aeruginosa* biofilm [236].

## 5. QQ Applications

In concert with the progress of knowledge regarding the molecular basis of the QQ process, innovative applications of QS inhibitors are providing encouraging results in mitigating the negative impact that microorganisms have in different fields such as agriculture, aquaculture, waste treatment, and human health.

Bacterial soft rot is a most pervasive and economically pernicious disease that affects a wide range of agricultural and horticultural crops and is caused by multiple genera of Gram-positive and Gram-negative phytopathogens [237]. In exploring pesticide-substitutive and environmentally friendly strategies that may finally allow for effective disease management, a QQ-based approach, mainly based on the identification of AHL-degrading strains, is gaining considerable attention. The *Pseudomonas segetis* strain P6 isolated from the halophyte plant *Salicornia europaea* displays biocontrol ability against the phytopathogens *Dickeya solani*, *Pectobacterium atrosepticum*, *P. carotovorum* subsp. carotovorum, and *Pseudomonas syringae* infecting tomato and carrot plants by degrading AHL [238]. *Bacillus thuringiensis* strains possess the AHL-lactonase AiiA, which suppresses the QS-dependent virulence of the plant pathogen *E. carotovora*, thereby reducing its pathogenicity and the incidence of the symptom development of potato soft rot [239]. Zang and coll. identified a highly efficient AHL-degrading *Pseudomonas nitroreducens* strain W-7 that is capable of degrading a wide range of AHLs, including N-(3-oxohexanoyl)-l-homoserine lactone (OHHL), N-(3-oxooctanoyl)-l-homoserine lactone (OOHL), and N-hexanoyl-l-homoserine lactone (HHL). It follows that this strain is a useful biocontrol agent against various bacterial phytopathogens [240]. Bacterial strains capable of degrading the Diffusible Signal Factor (DSF) involved in the regulation of pathogenic virulence have also been identified. Among these, we mention *Acinetobacter lactucae* strain QL-1, *Burkholderia anthina* strain HN-8, and *Burkholderia* sp. F25, characterized for their degradation ability and potential biocontrol of black rot disease caused by *Xanthomonas campestris* [241,242,243]. In addition to this biocontrol approach, transgenic plants modified with QQ lactonase have also been developed to prevent bacterial infection. Dong and coll. first demonstrated that tobacco and potato plants expressing aiiA lactonase from *Bacillus* sp. quench pathogen QS signaling and show significantly enhanced resistance to *E. carotovora* infection [71]. *Nicotiana tabacum* lines expressing the lactonase AttM efficiently quench *P. carotovorum* communication in vivo [244].

Stress conditions (due to overcrowding) and water quality in aquaculture make fish particularly susceptible to bacterial infections, mainly from *Vibrio*, *Pseudomonas*, *Edwardsiella*, *Flavobacterium*, and *Aeromonas*. In the last few years, non-conventional methods for controlling bacterial diseases and improving the general health conditions of cultured species have been adopted to attenuate the virulence of fish pathogens. Lactonases purified from *Bacillus* sp. can attenuate *A. hydrophila* infections in zebrafish [245]. The AiiA lactonase isolated from *Bacillus licheniformis* DAHB1 inhibits in vitro biofilm formation in *Vibrio* and reduces its colonization and the mortality of shrimps in aquaculture [246]. Finally, *Bacillus* sp. QSI-1, *B. licheniformis* T-1, *B. thuringensis* QQ1, *B. cereus* QQ2, and *Bacillus velezensis* D-18 significantly reduce the pathogenicity of *A. hydrophila* and biofilm formation in *Vibrio* sp. by downregulating the production of the respective virulence factors, thereby making them safe and effective for protecting hosts against pathogenic bacterial infections in aquaculture [247,248,249,250].

Membrane bioreactors (MBRs) represent the technology of choice to treat both industrial and municipal wastewaters. However, the accumulation of microbial cells and extracellular polymeric substances, namely, biofouling, represents the critical point that negatively impacts the filtration performance of the membranes. Therefore, the current non-chemical strategies for reducing membrane biofouling consist of the immobilization of QQ bacteria, QQ enzymes, or QQ natural compounds such as curcumin, flavonoids, and funarones on different surfaces to significantly inhibit biofilm formation [251,252,253].

Biofilm formation by pathogenic bacteria on medical devices and implants generally evolves into persistent and often recurrent infections that are often refractory to conventional antibiotic treatments and, therefore, challenging to treat. As elaborated upon in the previous paragraph, nanomaterials per se or in combination with QS inhibitors represent an innovative and effective solution to prevent, delay, and eradicate biofilm formation on surfaces or for enhancing drug targeting.

## 6. Conclusions

Currently, novel effective therapeutical approaches are being feverishly searched for to deal with the emergence and spread of antibiotic-resistant pathogens. In this context, a better characterization of the molecular mechanisms underlying QS and its inhibition, as well as the discovery of new compounds with quenching activity, turns out to be fundamental for the development of innovative and antibiotic-free multifaceted biocontainment approaches in different fields such as the clinical, food, and environmental agriculture fields.

## Figures and Tables

**Table 1:** Quorum Quenching enzymes, their substrates, and their relative mechanisms of inhibition of biofilm.

Enzyme Category	Name	Source	Substrates	Effect	Reference
Lactonases	AiiA	*Bacillus* sp. *Priestia aryabhattai*	C4-, C6-, C8-, C10-HSL; 3OC4-, 3OC6-, 3OC8-, 3OC12-HSL; 3-OH-C4-HSL	Inhibition of biofilm and production of pyocyanin, rhamnolipid, and exopolysaccarides in *P. aeruginosa*, *Vibrio cholerae*, and *S. aureus*.	[36,37]
	AiiB	*Agrobacterium tumefaciens*	C4-, C6-, C7-, C8-, C10-HSL; 3OC6-, 3OC8-HSL	Reduction in the bacterial virulence in *Erwinia carotovora*.	[38]
	AiiK	*Lactobacillus casei*, *Kurthia huakui*	C10-HSL	Attenuation of swimming motility, virulence factor production, and biofilm formation in *Aeromonas hydrophila* and *P. aeruginosa*	[39,40]
	Aii810	Bacteria from Mao-tofu	3OC12-HSL	Inhibition of virulence and biofilm in *P. aeruginosa*.	[41]
	bpiB01, B04, B07	Bacteria from soil samples	3OC8-HSL; 3OC6-, 3OC8-, 3OC12-HSL	Biofilm inhibition in *P. aeruginosa*.	[42]
	DlhR, QsdR1	*Rhizobium* sp.	3OC8-HSL	Inhibition of biofilm and other QS-dependent processes in *P. aeruginosa*, *Chromobacterium violaceum*, and *A. tumefaciens*.	[43]
	ND	*Geobacillus kaustophilus*	3-OH-C10-HSL, 3-OH-C12-HSL	Distruption of biofilm on multiple strains of *A. baumannii*.	[44]
	MCP	*Mycobacterium avium*	C6-, C7-, C8-, C10-, C12-HSL	The enzyme is a metal-dependent N-acylhomoserine lactonase	[45]
	MomL	*Muricauda olearia*	C6-HSL	Attenuation of the virulence of *P. aeruginosa*.	[46]
	SsoPox-1	*Sulfolobus solfataricus*	C4-, C6-, C8-, C12-HSL; 3OC6-, 3OC8-, 3OC10-, 3OC12-HSL	Attenuation of QS signaling, virulence factor production, and biofilm formation in vitro.	[47]
	PON1-3	*Homo sapiens*	C7-, C12-, C14-HSL; 3OC6-, 3OC10-, 3OC12-HSL	Inhibition of biofilm formation and extracellular virulence factors in *P. aeruginosa*.	[48]
	YtnP	*Stenotrophomonas maltophilia*	3O-C12-HSL	Inhibition of biofilm formation, induction of biofilm decomposition, and reducetion of extracellular virulence factors production.	[49]
Acylases	Aac	*Ralstonia solanacearum*	C7-, C8-, C10-HSL; 3OC8-HSL	Inhibition of QS mechanism in *C. violaceum*.	[50]
	AhlD	*Arthrobacter* sp.	OHHL, OHL and OdDH	Reduction in the AHL amount and pectate lyase activity in *Erwinia carotovora*.	[51]
	AhlM	*Streptomyces* sp.	C6-, C8-, C10-HSL; 3OC6-, 3OC8-, 3OC12-HSL	Decrease of the production of virulence factors, including elastase, total protease, and LasA, in *P. aeruginosa*.	[52]
	aibP	*Brucella melitensis*	3OC8-, 3OC12-HSL	Decrease of endogenous AHL accumulation within *Brucella melitensis*.	[53]
	AiiD	*Ralstonia* sp.	OC6-, OC8-, OC10-, OC12-HSL	QQ activity in *P. aeruginosa* achieved through an Acyl-homoserine lactone acylase hydrolyses the AHL amide, releasing homoserine lactone and the corresponding fatty acid.	[54]
	AiiO	*Ochrobactrum* sp. *A44*	3OC4-14-HSL	Inhibition of the virulence in *Pectobacterium carotovorum*.	[55]
	GqqA	*Komagataeibacter europaeus*	C8-, C10-, C12-HS	Inhibiton of biofilm formation.	[56,57]
	HacA, B	*Pseudomonas syringae*	C6-, C8-, C10-, C12-HSL; OC8-, OC10-, OC12-, OC14-HSL	Inhibition of biofilm formation, and colony morphology.	[58]
	MacQ	*Acidovorax* sp.	C6-, C8-, C10-, C12-HSL; 3OC8-HSL; OC6-, OC8-, OC10-, OC12-, OC14-HSL	Interference with the QS system.	[59]
	PA2385	*P. aeruginosa*	3COC12-HSL	QQ activity achieved through an N-acyl-homoserine lactone acylase	[60]
	PvdQ	*P. aeruginosa*	C8-, C10-, C12-HSL; OC12-HSL	Attenuation of *P. aeruginosa* virulence.	[61,62]
	QuiP	*P. aeruginosa*	C8-, C10-, C12-HSL; OC12-HSL	Gene expression regulation of QS systems in *P. aeruginosa*.	[63]
	Slac1, 2	*Shewanella loihica-PV4*	3OC10-HSL	Exhibition of in vitro QQ activity.	[64]
Oxidoreductases	BdcA	*E. coli*	3OC6-HSL	Dehydrogenase/reductase activity.	[65]
	BpiB05	Bacteria from soil sample	3-oxo-C8-HSL	Exhibition of in vitro QQ activity.	[66]
	BpiB09	Bacteria from soil sample	3-oxo-C8-HSL	Reduction in pyocyanin production, decreased motility, poor biofilm formation in *P. aeruginosa*.	[67]
	CYP102A1	*Bacillus megaterium*	C12-20-HSL (ω-1, ω-2, ω-3 hydroxylated)	In vitro oxidation of AHLs and their lactonolysis products acyl homoserines.	[68]
	ND	*Rhodococcus erythropolis*	3-oxo-AHL, N-(3-oxo-6-phenylhexanoyl) homoserine lactone, 3-oxododecanamide, n-(3-oxododecanoyl)-L-homoserine lactone	Exhibition of AHL oxidoreductase and amidolytic activities.	[69]
	QQ-2	Bacteria from water sample	3-oxo-C6-HSL; 3,4,4-trihydroxy-2-pentanone-5-phosphate	Reduction of AHL and AI-2 in QS-inactive hydroxy-derivatives and inhibits biofilm formation in *Klebsiella oxytoca* and *Klebsiella pneumoniae*.	[70]

**Table 2:** Quorum Quenching natural compounds and their relative molecular effects on biofilm.

Phytochemical Class	Compounds	Natural Source	Molecular Effect	Reference
Alkaloids	Berberine	*Coptis, Hydrastis* and *Berberis* genus	Downregulation of QS-related genes.	[111]
	Chelerythrine	*Chelidonium majus* L.	Disruption of membrane integrity, inhibition of biofilm components.	[112]
	Furocoumarins	Grapefruits	Inhibition of QS signalling; Biofilm dispersion.	[113,114]
	Norbgugaine	*Arisarum vulgare*	Inhibition of cell motility and of adhesion.	[115]
Anthraquinones	Emodin	*Rheum palmatum* L.	Suppression growth, hyphal development, and biofilm of *Candida albicans* by targeting cellular kinase signalling.	[116]
		*Polygonum cuspidatum, Rheum palmatum*	Downregulation of biofilm-forming related genes in *S. aureus*.	[117]
Glycosides	β-sitosterol glucoside; Isolimonic acid; Ichangin	*Citrus* species	Inhibition of the biofilm and motility through the repression of flagellar master operon flhDC Interferece with cell–cell signalling and biofilm formation by the modulation of luxO expression.	[118,119]
	Ellagic acid	*Rubus ulmifolius*	Inhibition of the biofilm and increase antibiotic susceptibility.	[120]
Lectins	AGL	*Amaranthus gangeticus*	Inhibition of biofilm formation.	[121]
	CasuL	*Calliandra surinamensi*	Inhibition of biofilm formation.	[122]
	ConA	*Canavalia ensiformis*	Reduction in bacterial adhesion.	[123]
	ConBol	*Canavalia boliviana*	Reduction in bacterial adhesion.	[124]
	ConM	*Canavalia maritima*	Reduction in bacterial adhesion.	[124]
	PgTeL	*Punica granatum*	Reduction in bacterial adhesion.	[125]
Polyacetylenes	Glicosides (PAGs)	*Launaea capitata*		[126]
Polyphenols	Casbane diterpene	*Croton nepetaefolius*	Inhibition of biofilm and reduction of bacterial adhesion.	[127]
	7-epiclusianone	*Rheedia brasiliensis*	Inhibition of biofilm and reduction of bacterial adhesion.	[128]
	Flavonoids (baicalin, catechin, curcumin, kaempherol, luteolin, proaminophyllin, quercetin, vitexin)	*Scutellaria baicalensis and Vitex* species	Decrease in the QS signaling molecules 3-oxo-C12-HSL and C4-HSL; Attenuation of LasA, LasB, and LuxR.	[129,130,131]
	Methyl-gallate		Suppression of the synthesis of the extracellular polymeric matrix, inhibition of QS signaling, and alteration of the microbial cell membrane.	[132]
	Phloretin	Apple, pear and strawberry fruits	Downregulation of virulence genes essential for surface attachment, biofilm formation, and QS.	[133]
Terpenoids	Carvacrol	*Organum vulgare*	Reduction in QS signalling.	[134]
	Ursolic acid	Many medical plants	Inhibition of biofilm formation and disruption of cell membrane.	[135]
	4-epi-primaric acid	*Aralia cachemirica* L.	Inhibition of biofilm formation and disruption of cell membrane.	[136]
